# Flatulent Colic

**Published:** 1895-05

**Authors:** Otto Noack

**Affiliations:** Reading, Pa.


### FLATULENT COLIC—POST-MORTEM.

BY OTTO NOACK, READING, PA.

*Colica.* Bay horse, showing the symptoms of severe flatulent colic. Died two hours afterward. Dissection on next morning. Animal was in poor condition. Upon opening the cavity of abdomen the pointed end of the cæcum was found in the left side of flank. The left part of the large colon was twisted around the longitudinal axis and around the diametrical axis. The mesentery of the small intestines was twisted at the commencement. The cæcum was of a bluish red color. Upon opening the intestines all the tissue of the small ones were of a brownish red color, the whole length was covered with diphtheritic coating, the color being a grayish green. The tissues of cæcum and first portion of large colon were swollen, and many hemorrhages were seen there. Upon cutting the stomach open the pylorus was swollen, grayish green and anæmic, the structure around the different glands lobuli appeared to be drawn in.

The liver looked glossy and smooth, grayish red in color, enlarged, rims curled. The parenchyma on the cut surface appeared grayish yellow and abnormally soft. The kidneys could be easily lifted out of capsules, were smooth and glossy, of tough consistence. Cutting diametrically the renal substance, it appeared to be bulged out, and was of a yellowish color. The straight urinary tubes were distended, and upon pressure a slimy grayish green fluid escaped. The spleen enlarged, capsules distended, steel blue in color and the pulp soft and flabby. The meshwork beneath it could not be distinguished plainly.

Heart muscle gray and soft. On the diametral cut surface of the lungs there was a froth resembling soapsuds.

The ligamenta aryepiglottica of the larynx were enlarged and thickened.

*Anatomical Diagnosis.* Implication of cæcum, longitudinal twisting of the left part of the large colon around the transverse colon, twisting of mensentery of small intestines, jejunitis hæmorrhagica et diphtherica, cholitis et typhlitis hæmorrhagica, gastritis glandularis, hepatitis parenchymatosa, nephritis catarrhalis, intumescentia lienis, myocarditis parenchymatosa, carditis, œdema pulmonum.

